# Replacing Dietary Fish Meal with Defatted Black Soldier Fly (*Hermetia illucens*) Larvae Meal Affected Growth, Digestive Physiology and Muscle Quality of Tongue Sole (*Cynoglossus semilaevis*)

**DOI:** 10.3389/fphys.2022.855957

**Published:** 2022-04-13

**Authors:** Xueting Li, Chuanjie Qin, Zhenzhen Fang, Xueliang Sun, Hongyue Shi, Qingkui Wang, Honghao Zhao

**Affiliations:** ^1^ Department of Fisheries, Tianjin Agricultural University, Tianjin, China; ^2^ Key Laboratory of Sichuan Province for Fishes Conservation and Utilization in the Upper Reaches of the Yangtze River, Neijiang Normal University, Neijiang, China

**Keywords:** fish meal substitution, *Hermetia illucens*, *Cynoglossus semilaevis*, growth performance, physiology and biochemistry

## Abstract

For solving the global shortage of fish meal (FM) supplies from fisheries, the black soldier fly (*Hermetia illucens*) has become a new protein alternative in aquatic feeds. The present study investigated the effects of dietary inclusion of defatted *H. illucens* larvae meal (DBLM) on growth, serum biochemical parameters, digestive function, and muscle quality of tongue sole (*Cynoglossus semilaevis*). The feeding experiment consisted of five experimental diets: a control diet based on FM protein (H0) and four DBLM diets, substituting 25% (H25), 50% (H50), 75% (H75), and 100% (H100) of FM. *C. semilaevis* (initial weight 563.48 ± 22.81 g) were randomly allocated over five treatments in quadruplicate. After 65 days of feeding, the weight gain rate (WGR), specific growth rate (SGR), and protein efficiency ratio (PER) were significantly higher in H0 and H25 groups with less feed conversion ratio (FCR) and feed intake (FI). The concentrations of serum ALT, TG, T-CHO, ALB, and GLO and their ratio (i.e., A/G) in the H25 group were also significantly higher than those in the other DBLM diet-feeding groups. The digestive enzyme activities first increased (from 25% to 75%) and then decreased (from 75%) with the increased level of DBLM in diets. Meanwhile, there were significant improvements in the thickness of the intestinal longitudinal muscle (LM), circular muscle (CM), columnar epithelium (CE), and lamina propria (LP) in H25 *C. semilaevis* compared to the control group (*p* < 0.05). The fish from the other DBLM diets groups presented significant reductions in the thicknesses of LM, CM, CE, and LP, as well as the length of microvilli (ML) in a dose-dependent manner (*p* < 0.05). However, the substitution of FM increased up to 50% would result in intestinal structural damage. Moreover, the proximate compositions, antioxidant and water holding capacity, and muscular structures of *C. semilaevis* fillets were all significantly affected after substituting 25% FM with DBLM (*p* < 0.05). Except for the dry matter, moisture, ash, crude fat, and protein contents were significantly higher in H25 *C. semilaevis* muscles. The SOD activity in the H0 group was significantly lower than that in the H25 group. The CAT activity in *C. semilaevis* muscles prominently reduced along with the increase in DBLM content in feeding diets (*p* < 0.05). The water holding capacity of *C. semilaevis* fillets was best in the H25 group. In summary, the optimum proportion of DBLM with FM for feeding *C. semilaevis* may be around 25%.

## Highlights

(1) Replacing 25% of the fish meal with defatted black soldier fly (*Hermetia illucens*) larvae meal (DBLM) had positive effects on the weight gain rate, specific growth rate, condition factor, and survival rate of *C. semilaevis* while decreasing the feed conversion ratio and feed intake;
(2) Feeding *C. semilaevis* with DBLM diets brought the serum T-CHO and TG down and relieved the fat deposition in the fish body. Substituting fish meal with DBLM up to 50% resulted in intestinal structural damage and abnormal liver function;
(3) The replacement level of less than 75% could boost the antioxidant capacity of *C. semilaevis* muscles. The water holding capacity was the best in the H25 *C. semilaevis* fillets.

## 1 Introduction

It is well recognized that the increasing demand and unstable fish meal production have led to an increase in the cost of aquaculture production. The substitution of high-value and unsustainable fish meal (FM) by less expensive and more readily available alternatives in aquatic feeds is a priority for the sustainable development of the aquaculture sector (Jeong et al., 2021a). Therefore, it is of practical significance to replace FM with cheaper, protein-rich animal and plant ingredients (Tacon and Metian, 2008; Hardy, 2010). The growing empirical evidence has suggested that the insect meal has higher nutritive value, greater feed conversion efficiency, extremely smaller environmental footprint, and relatively lower price than FM and other alternative protein sources (Wang and Shelomi, 2017; Nogales-Mérida et al., 2019; Smets et al., 2020). Meanwhile, partially replacing FM with black soldier fly (*Hermetia illucens*) meal is highly feasible for most marine and freshwater fish species (Xu et al., 2020). Nevertheless, the extent to which FM can be spared by *H. illucens* meal without negative consequence depends on several factors, including fish species, life stages, and the differed processing technologies of *H. illucens*, as well as feeding strategies (Magalhães et al., 2017; Smets et al., 2020; Takakuwa et al., 2021).

*Hermetia illucens* can convert low-value organic waste into valuable fat- and protein-rich biomass (Raksasat et al., 2020). It has been testified that the extraction of lipid, protein, amino acid, and fatty acid profiles might differ among the developmental stages (larvae, prepupae, and pupae) of *H. illucens*. Especially, the most balanced and higher nutrients were measured in larvae (Smets et al., 2020). Specifically, their larvae are 38.9%∼59.8% crude protein and 29%∼41% fat, and the essential amino acid (EAA) profile is very similar to FM (Nogales-Mérida et al., 2019; Smets et al., 2020). When defatted, *H.* illucens larvae meal (DBLM) can have crude protein levels over 60% and more abundant nutrients (Spranghers et al., 2017; Wang and Shelomi, 2017). In addition, the total amount of EAA in DBLM was about 3 times that of FM; lauric acid (C12:0) is almost 2.6 times higher than that in FM; and the antimicrobial peptide was also identified in DBLM (Lee et al., 2020; Tippayadara et al., 2021). Therefore, DBLM has higher potential and more advantages to replace FM in aquatic feeds than either full-fat or partially defatted *H. illucens* larvae meal (Renna et al., 2017; Rawski et al., 2020).

The DBLM partial replacement of FM in diets has been successfully reported in several fish species. For example, the feed conversion ratio (FCR), total saturated, and monounsaturated fatty acids (SFA and MUFA) increased significantly at each increment level of DBLM, while polyunsaturated FA (particularly n-3 FA) decreased. Meanwhile, the DBLM diets resulted in similar apparent digestibility of nutrients, growth performances, proximate composition, and histomorphology of *Oncorhynchus mykiss* compared with the control group (Renna et al., 2017; Caimi et al., 2021). Li et al. (2017) confirmed that the DBLM diets had no significant effects on *Cyprinus carpio* growth, nutrient utilization, and digestive enzyme activity. However, the DBLM diets boosted the antioxidant capacity of *C. carpio* by strengthening the CAT activity and reduced cholesterol content and lipid deposits in tissues. Thus, it is suitable to replace up to 50% of FM with DBLM for *C. carpio*; the dietary stress and intestinal histopathological damages were observed if the replacement levels exceeded 75% (Li et al., 2017). In *Perca fluviatilis* research, reduced fatty acid content and n-3/n-6 ratio were found with increasing DBLM inclusion, whereas the higher specific growth rate was tested in the control and 20% and 40% DBLM groups. Therefore, 40% inclusion of DBLM can be used successfully in standard diets for *P. fluviatilis* (Stejskal et al., 2020). The abovementioned similar findings were confirmed once again in *Danio rerio*, which also revealed the positive impacts of DBLM on fish at the molecular level (Lanes et al., 2021). In general, the DBLM not only has high nutritive value but also represents good palatability, characteristics that can be better absorbed and utilized, and some beneficial effects on farmed fish. However, it is still blank for the application of DBLM in tongue sole (*Cynoglossus semilaevis* Günther) diets and its improving effect on fish flesh quality.

*Cynoglossus semilaevis* is distributed in Chinese coastal waters, particularly abundant in the northern region, such as off Tianjin and Shandong peninsula (Lin et al., 2021). This kind of fish is a promising species for aquaculture and a popular commercial flatfish in China due to its high nutritive value and pleasant taste. Intensive farming based on the utilization of artificial feeds has contributed to the continuous growth in the production of *C. semilaevis*. Unfortunately, the fish flesh quality declined over time through the course of artificial aquaculture based on artificial feeds (rich in FM protein) feeding (Valente et al., 2013). Searching for alternative proteins for FM and obtaining high-quality fish products are the key targets for a successful aquaculture industry in the future. To fulfill this aim, we conducted a comprehensive study to compare the effects of DBLM diets and artificial feeds on the growth, digestion, muscle characteristics, and quality of *C. semilaevis*. The results would provide a theoretical basis and reference for further research on the wide application of DBLM in aquatic feeds and improving the flesh quality of aquatic products fed with DBLM diets.

## 2 Methods and Materials

### 2.1 Experimental Diets and Proximate Composition

The primary dietary ingredients of conventional *C. semilaevis* feeds are as follows: super steam fish meal (FM), defatted *H. illucens* larvae meal (DBLM), fermented soybean meal, shrimp meal, and fish oil. The five isonitrogenous and isolipidic experimental diets were formulated by substituting 0%, 25%, 50%, 75%, and 100% FM protein with DBLM (H0, H25, H50, H75, and H100, respectively). The composition and proportion of other components in experimental diets were summarized in Table 1 (fish oil, high gluten flour, and microcrystalline cellulose contents were different in order to regulate the lipid and protein contents in each group’s feeding diet).

**TABLE 1:** Ingredients and proximate composition of the experimental diets.

Ingredients (g/kg)	Experiment diets				
	H0^a^	H25	H50	H75	H100
Super steam fish meal	50.00	37.50	25.00	12.50	0.00
Defatted *H. illucens* larvae meal	0.00	14.41	28.81	43.22	57.63
Fermented soybean meal	13.50	13.50	13.50	13.50	13.50
Broken yeast	4.00	4.00	4.00	4.00	4.00
Shrimp meal	6.00	6.00	6.00	6.00	6.00
Gluten	4.47	4.20	3.90	3.67	3.37
Fish oil	5.90	7.00	8.10	9.20	10.30
Microcrystalline cellulose	11.77	8.83	5.93	2.98	0.07
Calcium dihydrogen phosphate	1.40	1.40	1.40	1.40	1.40
Multivitamins^b^	1.50	1.50	1.50	1.50	1.50
Complex minerals^c^	0.10	0.10	0.10	0.10	0.10
50% choline chloride	1.00	1.00	1.00	1.00	1.00
Lysine	0.00	0.15	0.30	0.42	0.55
Methionine	0.00	0.05	0.10	0.15	0.22
Sodium polyacrylate	0.30	0.30	0.30	0.30	0.30
Clomerin	0.03	0.03	0.03	0.03	0.03
Ethoxyquinoline dry powder	0.03	0.03	0.03	0.03	0.03
Total	100.00	100.00	100.00	100.00	100.00
**Proximate composition (%)**					
Crude fat	10.10	10.50	10.20	10.40	10.90
Crude protein	56.55	56.62	53.91	53.93	52.46
Ash	5.64	5.85	5.93	5.12	4.82
Moisture	10.77	12.92	13.50	16.30	15.88

a H0 = 100% super steam fish meal; H25 = 25% DBLM; H50 = 50% DBLM; H75 = 75% DBLM; H100 = 100% DBLM.

b Mineral premix (g/kg of mixture): Ca(H_2_PO_4_)_2_·H_2_O, 14; ZnSO_4_·7H_2_O, 0.065; CuSO_4_·5H_2_O, 0.01; FeSO_4_·7H_2_O, 0.109; MnSO_4_·H_2_O, 0.024; KI, 0.032; CoCl_2_·6H_2_O, 0.0002; Na_2_SeO_3_, 0.0003; zeolite powder, 5.76.

c Vitamin premix (g/kg of mixture): V_A_ 0.03; V_D3_ 0.01; nicotinamide 0.25; inositol 1.00; calcium pantothenate 0.06; V_B1_ 0.01; V_B2_ 0.05; V_B6_ 0.02; V_B12_ 0.012; folic acid 0.015; V_E_ 0.40; V_C_ 0.60; V_K3_ 0.005; biotin 0.06; wheat middling 5.133.

Chemical analyses of the experimental diets were analyzed according to AOAC methods (AOAC, 2012). The samples were dried to a constant weight at 105°C for 24 h to determine the dry matter content. Then, the crude protein was determined by the Kjeldahl method after acid digestion. The percentage of chitin and its nitrogen content is not considered. Crude fat was carried out according to Folch et al.’s method (Folch et al., 1957). The ash content was determined using incineration in a muffle furnace at 550°C for 12 h (ISO 5984-2002). The proximate compositions of the five experimental diets are given in Table 1.

### 2.2 Experimental Fish and Feeding Trial

*Cynoglossus semilaevis* (mean initial weight 563.48 ± 22.81 g) was provided by Haisheng Aquaculture Co., Ltd. (Tianjin, China). The experimental fish were randomly distributed into 20 tanks (80 L) with *n* = 5 fish per tank and acclimatized to the feeding experimental conditions for 7 days. The feeding trials were conducted in an indoor recirculating system. The fish were fed with an experimental diet of the corresponding experimental group prior to the formal feeding experiment, twice a day at 9:00 and 19:00. Each tank was outfitted with a continuous aeration device. During the whole feeding period, the water temperature was maintained at 17∼20°C. The dissolved oxygen in the water was controlled above 7 mg/L, ammonia-N less than 0.01 mg/L. For 65 days, *C. semilaevis* were fed to apparent satiation, at 9:00 and 19:00, with a daily feeding rate of 0.5% of body weight. The dead fish were weighed and their amount in each group was recorded, respectively.

### 2.3 Sample Collection and Analysis

After 24 h starvation, five fish from each tank were anesthetized by 100 mg L^−1^ of MS-222 (Sigma, St. Louis, MO, United States). The growth performances (including final body weight—FBW, body length, and body height) were measured and calculated from a total of 20 tails of fish per group before slaughter. In each tank, three individuals collected blood samples from the caudal vein using 2 ml syringes, the separated serum was removed by centrifuging (3,500 r/min, 10 min) after keeping it at 4°C for 12 h. Then, the separated serum was stored at −80°C until the serum biochemical analysis. After blood sampling, all of the experimental fish were immediately sampled liver, intestine, and muscle tissues, then frozen in liquid nitrogen. All these samples were transferred and preserved at –80°C until subsequent detection.

Afterward, the instantaneous growth rate (IGR), specific growth rate (SGR), weight gain rate (WGR), feed conversion ratio (FCR), protein efficiency ratio (PER), condition factor (ConF), feed intake (FI), survival rate (SR) were calculated via the following formulas (Crane et al., 2020):

$$\begin{array}{l} \text{IGR }\left(\text{\%}\right)\;=\;\frac{\text{Ln }\left({\text{W}}_{\text{f}}\right)\;-\text{ Ln }\left({\text{W}}_{\text{i}}\right)}{\text{T}};\\ \text{SGR }\left(\text{\%}\right)\;=100\left(e^{IGR}-1\right); \end{array}$$ (1)

$$\text{WGR }\left(\text{\%}\right)\;=\;\frac{{\text{W}}_{\text{f}}{\;-\text{ W}}_{\text{i}}}{{\text{W}}_{\text{i}}}\text{ × }100\text{\%;}$$ (2)

$$\text{FCR }=\frac{{\text{W}}_{\text{0}}}{{\text{W}}_{\text{f}}{\;-\text{ W}}_{\text{i}}};$$ (3)

$$\text{FI }\left(\text{\%}\text{tail}\;\right)\;=\frac{{\text{W}}_{\text{F}}}{{\left({\text{W}}_{\text{f}}\;+\text{ W}\right)}_{\text{i}}/2}\times \;100\%;$$ (4)

$$\text{PER }=\;\frac{{\text{W}}_{\text{f}}{\;+\text{ W}}^{\text{′}}{\;-\text{ W}}_{\text{i}}}{\text{FI × P}}\text{ × }100\text{\%;}$$ (5)

$$\text{ConF }=\frac{{\text{W}}_{\text{f}}}{{\text{L}}^{3}}\text{× }100\text{;}$$ (6)

$$\text{SR }\left(\text{\%}\right)=\;\frac{{\text{N}}_{1}}{{\text{N}}_{2}}\text{ × }100\text{\%}\text{.}$$ (7)

Here, W_0_, amount of feeds given (g); W_f_, final body weight (g); W_i_, initial Weight (g); W’, weight of dead fish during the feeding period (g); W_F_, the total amount of the feed consumed (g); T, feeding days (65 d); P, protein content in the specific experimental feeds (%); L, body length (cm); N_1_, the final tails of fish in the specific experimental group; N_2_, the initial number of fish in the corresponding group.

This study complied with the Animal Research: Reporting of *In vivo* Experiments (ARRIVE) guidelines and the “Guidelines for Experimental Animals” from the Ministry of Science and Technology (Beijing, China). Furthermore, the Institutional Animal Care and Use Ethics Committee of Tianjin Agricultural University had approved our study. All efforts were made to minimize the suffering of sampled fish individuals.

### 2.4 Determination of Serum and Intestine Biochemical Parameters

The serum samples were prepared according to the previous method (Shi et al., 2006). The total protein (TP), albumin (ALB), globulin (GLO), aspartate aminotransferase (AST), alanine aminotransferase (ALT), triglyceride (TG), and total cholesterol (T-CHO) were tested using an automatic biochemistry analyzer (Hitachi 7020, Hitachi High Technologies, Inc., Ibaraki, Japan). The test kits were purchased from Nanjing Jiancheng Biochemical Corporation (Nanjing Jiancheng Biochemical Corporation, Nanjing, China).

In addition, the activities of trypsin and lipase in the intestines were determined using kits (Nanjing Jiancheng Bioengineering Institute, Nanjing, Jiangsu, China). The procedures followed were in accordance with the instructions of the kit.

### 2.5 Histological Analysis

The midgut (i.e., mid-intestine) was dissected and stored in 10% buffered formalin. The samples were processed according to the standard histological method in place at the Institute of Aquaculture (Stirling, United Kingdom). Briefly, the samples were dehydrated in ethanol, equilibrated in xylene, and embedded in paraffin. Transverse cuts of approximately 8 μm were stained with hematoxylin & eosin (H&E staining) (Rasmussen and Ostenfeld, 2000). Each slide was observed under a microscope. ImageJ was used to quantify the effects of DBLM diets on intestinal morphology (for example, lumen, columnar epithelium, lamina propria, goblet cells, stratum granulosum, circular muscle, longitudinal muscle, and serosa) of *C. semilaevis* (Urán et al., 2008; Cavrois-Rogacki et al., 2022)*.*

Serial transverse 8 µm-thick sections were stained with H&E according to the procedures (Rasmussen and Ostenfeld, 2000). A total of 200–400 fibers of white muscle per fish were studied using a Leica MZ 6 microscope for their cross-sectional area (CSA) and the diameter (*d* = 2*r*) of each fiber was calculated from the fiber area (A) [A = *π* × *r* ^2^, thus, *d* = 2√ (A × *π* ^−1^)]. A size limit for identifying the fibers was set at fiber diameters ≥10 µm since the optical resolution below this limit did not allow for sufficient identification and accuracy in the analyses (Luther et al., 1995). The circularity of each fiber (4*π* × A × circumference^−2^) was also determined. A circularity of 1.0 indicated a complete circle. The free software ImageJ (http://rsb.info.nih.gov/ij/) was also used for quantitative statistics and analyses.

### 2.6 Antioxidant Capacity and Oxidative Stress

For the analyses of antioxidant enzymes activity and oxidative stress, muscle samples were collected (three fish per tank) and used for determination. All the samples were flash-frozen in liquid nitrogen and stored at −80°C until follow-up analysis. Quality muscle samples were taken, the surface blood was washed with normal saline, and then, nine times the volume of 0.85% normal saline was added, homogenized in an ice water bath, and centrifuged at 4°C (2,500 r/min, 15 min). The supernatant was used for the determination of antioxidant enzymes, including superoxide dismutase (SOD), total antioxidant capacity (T-AOC), malondialdehyde (MDA), catalase (CAT), and lipid peroxide (LPO) contents with test kits (Nanjing Jiancheng Bioengineering Institute, China).

### 2.7 Measurement of Muscle Drip Loss, Frozen Exudation Rate, and Cooking Loss

Each fish took three parts of about 3.00 g peeled white muscle and weighed (W_1_, W_3_, and W_5_), which were stored at 4°C for 24 h, −20°C for 24 h, and put into a 5 ml centrifuge tube, respectively. After removing the muscle, wipe off the moisture from the surface, weigh (W_2_ and W_4_), and calculate the drip loss (DL) and the frozen exudation rate (FET). The centrifuge tube cover is cutoff, sealed with a sealing film, and a vent tied on the sealing film, which is put into a 75°C water bath for 15 min. Then, the moisture on the surface of muscle samples is absorbed, and subsequently weighed (W_6_). Afterward the DL, FET, and cooking loss (CL) were calculated based on the measured data.

$$\begin{array}{l} \text{DL }\left(\text{\%}\right)=\;\left({\text{W}}_{1}\;-{\text{ W}}_{2}\right)/\text{W}1\text{ × }100\text{\%};\left(8\right)\text{FET}\left(\text{\%}\right)\\ =\;\left({\text{W}}_{3}\;-{\text{ W}}_{4}\right)/{\text{W}}_{3}\text{ × }100\text{\%};\left(9\right) \end{array}$$
$$\text{CL}\left(\text{\%}\right)\;=\;\left({\text{W}}_{5}\;-{\text{ W}}_{6}\right)/{\text{W}}_{5}\text{× }100\text{\%}\text{.}$$ (10)

### 2.8 Statistical Analysis

The white muscle fiber circularity and intestinal morphology data were ln-transformed using software ImageJ. The fiber diameter was additionally fitted as a covariate to fiber circularity. For comparing the distributions of muscle fiber sizes, the non-parametric Kolmogorov-Smirnov two-sample test was applied. This test is very sensitive in detecting the differences in dispersions and skewness compared to other statistical tests (Zar, 1996). The ImageJ software was also used for intestinal morphometric analysis. One-way ANOVA (multiple comparisons) was used for analyzing the differences in the muscle characteristics and intestinal structures among groups. The differences were considered significant at *p* < 0.05 and highly significant at *p* < 0.01.

The data analyses of all test indicators (growth performances, biochemical indexes, and muscle drip loss, frozen exudation rate, and cooking loss) were presented as the “mean ± SE.” One-way analysis of variance was used to test the effect of diets. Duncan’s multiple range test was used for multiple comparisons. The 0.05 probability level was used to denote statistically significant data, while the 0.01 probability level was used to denote highly significant data.

## 3 Results

### 3.1 Growth Performances and Feeding Situations

The effects of experimental diets formulated by substituting different levels of FM protein with DBLM on survival, growth performance, and feed utilization of *C. semilaevis* were illustrated in Table 2. No significant differences were observed in the survival rate (SR) among the five groups, and the SR of *C. semilaevis* fed with DBLM diets remained higher (90%∼100%). The weight gain rate (WGR), specific growth rate (SGR), and protein efficiency ratio (PER) were significantly lower in H50, H75, and H100 groups than those in the control group (*p* < 0.05). However, the WGR, IGR, SGR, and PER in the H25 group were all significantly greater than those in H0 (*p* < 0.05). The less feed conversion ratio (FCR) was tested in the H0 and H25 groups; which were significantly different from that of H50, H75, and H100 groups (*p* < 0.05). Meanwhile, the greatest FCR was found in the H75 group. When replacing the FM with DBLM up to 50%, it had no significant effect on the feed intake (FI) of fish, and then, it significantly became enhanced in the H75 and H100 groups. Moreover, there was no significant difference in the condition factor (ConF) among different treatments (*p* > 0.05). Overall, most of the indices investigated the largest and optimal values in the H25 group, namely, the 25% DBLM could better promote the growth and feed utilization of *C. semilaevis*.

**TABLE 2:** Growth performances of *C. semilaevis* fed with different levels of DBLM (*n* = 20).

	H0	H25	H50	H75	H100
IW (g)	520.98 ± 10.39	522.79 ± 21.57	563.48 ± 22.81	588.47 ± 22.76	585.05 ± 21.37
FW (g)	686.75 ± 10.42	709.14 ± 21.06	699.67 ± 21.66	690.74 ± 22.23	722.22 ± 20.69
WGR (%)	31.86 ± 0.76^c^	35.84 ± 1.61^d^	24.31 ± 1.16^b^	17.46 ± 0.76^a^	23.55 ± 0.95^b^
FCR	1.26 ± 0.04^a^	1.14 ± 0.05^a^	1.61 ± 0.09^b^	2.34 ± 0.09^c^	1.75 ± 0.05^b^
IGR (%/d)	0.43 ± 0.01^c^	0.47 ± 0.02^d^	0.33 ± 0.01^b^	0.25 ± 0.01^a^	0.33 ± 0.01^b^
SGR (%)	1.66 ± 0.05^c^	1.97 ± 0.12^d^	1.16 ± 0.07^b^	0.77 ± 0.04^a^	1.12 ± 0.06^b^
PER	30.32 ± 0.16^c^	31.22 ± 0.12^d^	28.04 ± 0.11^b^	25.56 ± 0.19^a^	25.63 ± 0.17^a^
FI (%/d)	2.12 ± 0.04^a^	2.12 ± 0.01^a^	2.13 ± 0.06^a^	2.30 ± 0.01^b^	2.27 ± 0.02^b^
ConF (g/cm^3^)	0.77 ± 0.02	0.81 ± 0.03	0.79 ± 0.03	0.75 ± 0.04	0.75 ± 0.02
SR (%)	95.00 ± 5.00	100.00 ± 0.00	90.00 ± 5.77	100.00 ± 0.00	95.00 ± 5.00

Initial weight, IW; final body weight, FW; weight gain rate, WGR; feed conversion ratio, FCR; instantaneous growth rate, IGR; specific growth rate, SGR; protein efficiency ratio, PER; feed intake, FI; condition factor, ConF; survival rate, SR. The data measured traits of growth performances were presented as the “Mean ± SE.” Different lowercase letters mean the significant differences (*p* < 0.05) from each other.

### 3.2 Serum Biochemical Indices

**FIGURE 1:**
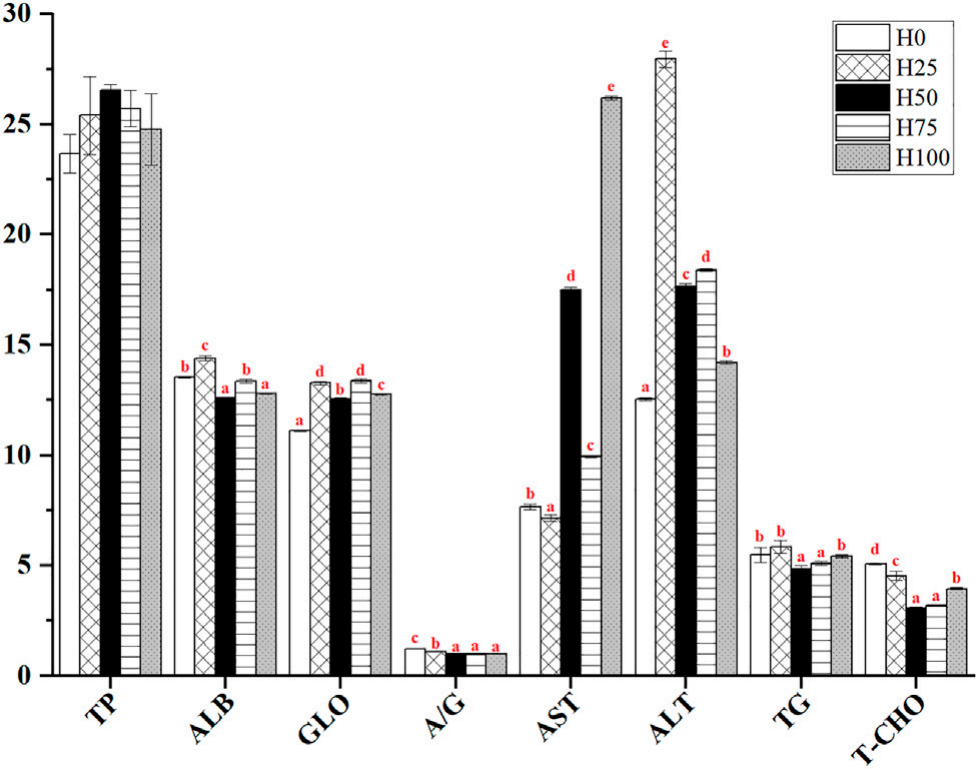
Serum biochemical parameters of *C. semilaevis* were fed with different levels of DBLM diets (*n* = 4). Total protein (TP, g/L), albumin (ALB, g/L), globulin (GLO, g/L), albumin/globulin ratio (A/G), aspartate aminotransferase (AST, U/L), alanine aminotransferase (ALT, U/L), triglyceride (TG, mmol/L), and total cholesterol (T-CHO, mmol/L). The data were expressed by “mean ± SE.” Different lowercase letters were used for presenting the significant differences among the five experimental groups, and significant at the 0.05 level.

As shown in Figure 1, no significant difference was found in the serum TP content for *C. semilaevis* among all the groups (*p* > 0.05). In the H25 group, the serum ALB, GLO and their ratio (i.e., A/G), as well as ALT, TG, and T-CHO contents were significantly higher than those of the other DBLM diet-feeding groups (including H50, H75, and H100 groups). The highest ALB, ALT, and TG levels were also detected in the same group. However, the comparison for each index between H25 and H0 groups showed complexity and index specificity. For instance, the levels of ALB, GLO, ALT, and TG in serum from the H25 group were all significantly higher compared to the H0 group, but the greater level of A/G and T-CHO were found in the control group (*p* < 0.05). For the AST activity, it was significantly inhibited in the H25 group, whilst exhibiting strengthened activity with the increase of FM replacement level. Accordingly, the greatest activity of AST was detected in the H100 group (*p* < 0.05) (Figure 1).

### 3.3 Intestinal Biochemical Indices

**FIGURE 2:**
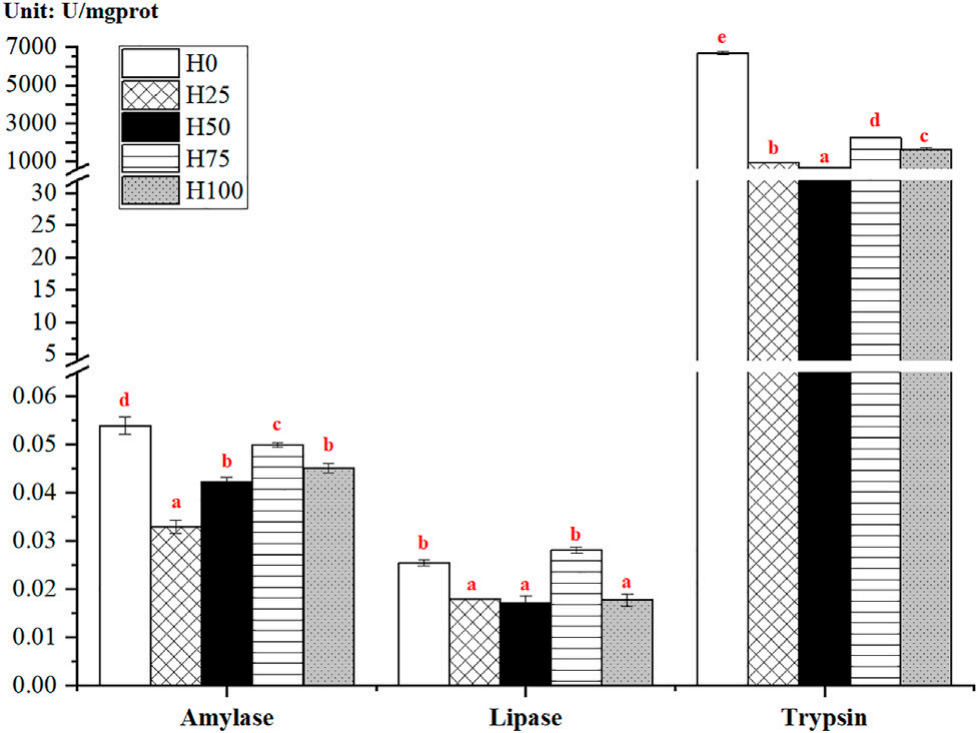
Activity of the intestinal digestive enzymes in *C. semilaevis* fed with experimental feeds substituting FM with different levels of DBLM (*n* = 4). The activities of intestinal amylase (U/mgprot), lipase (U/mgprot), and trypsin (U/mgprot) in *C. semilaevis* from five experimental groups, as well as their pairwise comparisons between groups. The significant differences between the groups were indicated by various red lowercase letters (*p* < 0.05).

*Cynoglossus semilaevis* fed with the DBLM diets (namely, from H25, H50, H75, and H100 groups) presented significantly lower activities of intestinal digestive enzymes than those in the control group (*p* < 0.05) (Figure 2). The highest levels of amylase and trypsin were measured in the H0 group. However, the greatest activity of lipase was observed in the H75 group, it showed no significant changes relative to the H0 group (*p* > 0.05). The comparisons between DBLM diet-feeding groups illustrated that the intestinal amylase, lipase, and trypsin activities had a similar altered trend with the increased substitution level of DBLM for FM in feeds. To be specific, the activity of digestive enzymes firstly increased (from 25% to 75%) and then decreased (from 75%) with the increased level of DBLM in diets. The lowest values of amylase, lipase, and trypsin were detected in the H25 and H50 groups, respectively (Figure 2).

### 3.4 Morphology of the *Cynoglossus semilaevis* Intestine

**FIGURE 3:**
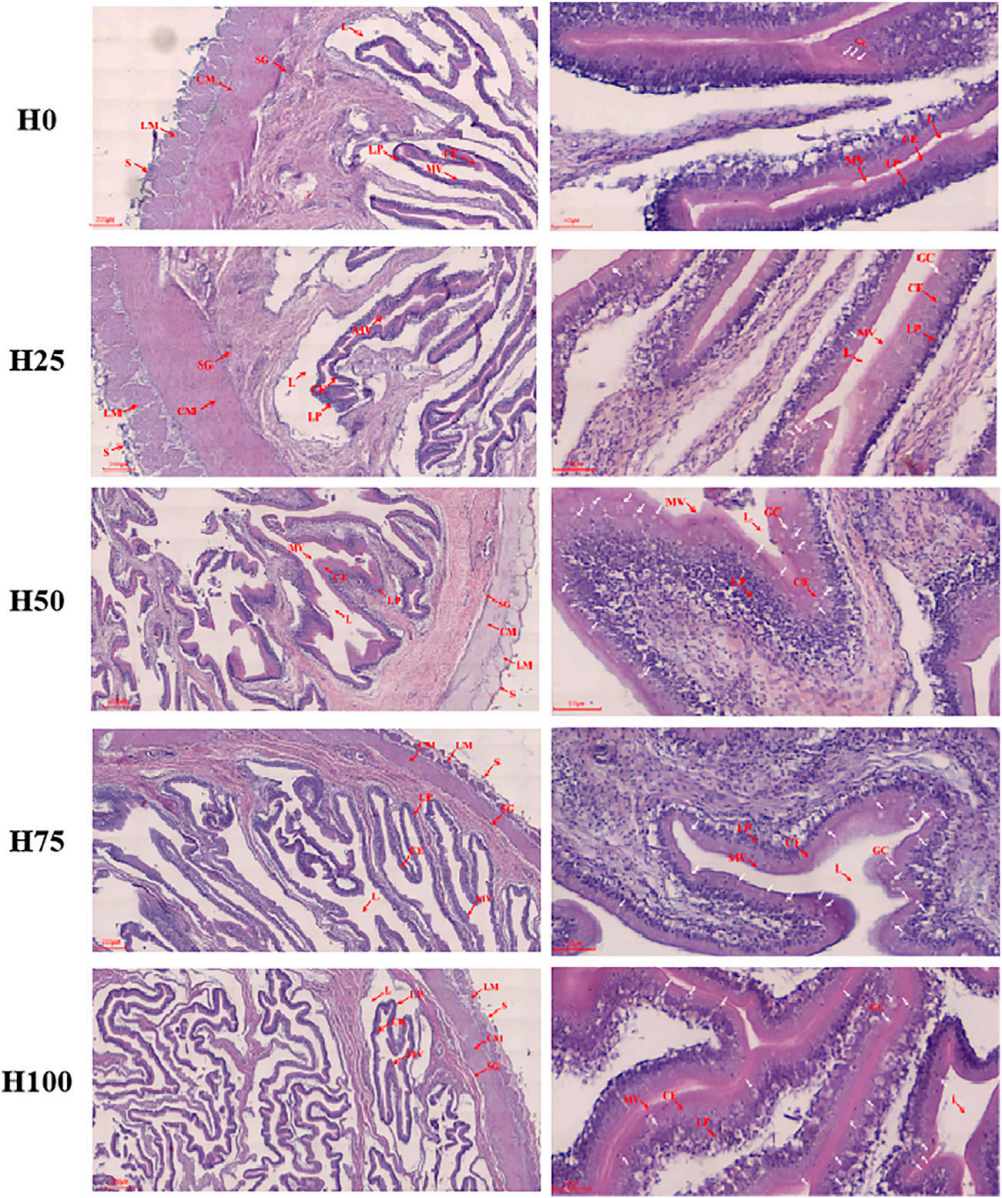
Midgut construction of *C. semilaevis* fed with the five different experimental diets. Transverse section of *C. semilaevis* mid-intestine (left column: ×40, right column: ×200) with structural organization. L, lumen; CE, columnar epithelium; LP, lamina propria; GC, goblet cells (multiple white arrow marked); MV, microvilli; SG, stratum granulosum; CM, circular muscle; LM, longitudinal muscle; and S, serosa.

The midgut construction of *C. semilaevis* was assayed in Figure 3; meanwhile, the statistical information of the data quantified from the tissue sections was supplied in Table 3. In the H25 group *C. semilaevis*, there were significant improvements in the thickness of intestinal longitudinal muscle (LM), circular muscle (CM), the columnar epithelium (CE), and lamina propria (LP) in comparison to the control group (*p* < 0.05). Whereas the fish from the other DBLM diets-feeding groups (i.e., H50, H75, and H100) presented significant reductions in thicknesses of LM, CM, CE, and LP, as well as the length of microvilli (ML) compared to the H0 and H25 groups, in a dose-dependent manner (*p* < 0.05). Thereby, the smallest CE, LP, and ML were observed in the H100 group. However, the LM and CM were thinnest in the H75 group, then they showed slight recoveries in the H100 group (Table 3). Furthermore, the DBLM diets also had significant effects on the goblet cell (GC). The numbers of GC increased with the increasing replacement level of FM (Figure 3). Moreover, the substitution level of the FM increased up to approximately 50%, accompanied by intestinal structural damage, such as splits in serosa and intestinal villus (Figure 3).

**TABLE 3:** Jejunum morphology of *C. semilaevis* in each experimental group.

	H0	H25	H50	H75	H100
Longitudinal muscle (μm)	125.86 ± 4.00^c^	188.82 ± 3.63^d^	60.59 ± 1.64^b^	43.90 ± 1.19^a^	57.35 ± 1.33^b^
Circular muscle (μm)	211.91 ± 4.76^d^	281.30 ± 3.06^e^	76.33 ± 2.96 ^ab^	70.26 ± 1.55^a^	83.54 ± 1.13 ^bc^
Columnar epithelium (μm)	27.57 ± 0.48^b^	32.38 ± 0.59^d^	37.55 ± 0.34^e^	29.74 ± 0.21^c^	24.91 ± 0.19^a^
Lamina propria (μm)	51.54 ± 0.46^c^	60.98 ± 0.81^e^	54.57 ± 0.37^d^	38.69 ± 0.24^b^	26.31 ± 0.21^a^
Microvilli length (μm)	31.00 ± 0.58^e^	19.34 ± 0.34^c^	22.41 ± 0.31^d^	8.19 ± 0.19^b^	6.99 ± 0.13^a^

### 3.5 The Comparison of the Muscle Characteristics of *Cynoglossus semilaevis* Between Groups

#### 3.5.1 Proximate Compositions of *Cynoglossus semilaevis* Flesh

Compared to the H0 group, moisture, ash, crude fat (CF), and protein (CP) contents of *C. semilaevis* flesh all significantly enhanced after substituting 25% FM with DBLM (*p* < 0.05). There were no significant alterations in moisture, DM, and ash contents along with the changes in substitute rate of DBLM (namely, from 25% to 100%). As for the CP, it significantly decreased with the increase of replacement level of FM with DBLM in feeds (*p* < 0.05). However, the CF in *C. semilaevis* muscles demonstrated complex changes among different feeding groups. It rose dramatically in the H25 group, decreased and then increased slightly in the H50 and H75 groups, declined sharply in the H100 group, respectively (*p* < 0.05). It is noteworthy that the higher levels of muscular moisture, ash, CF, and CP were found in the H25 group, while their lowest values were mostly detected in the either H0 group or the H100 group (Table 4).

**TABLE 4:** Proximate compositions of *C. semilaevis* muscles from the five experimental groups (% dry weight).

	H0	H25	H50	H75	H100
Moisture	79.57 ± 0.55^a^	80.92 ± 0.66^b^	80.08 ± 0.21 ^ab^	80.02 ± 0.17 ^ab^	80.21 ± 0.25 ^ab^
Dry matter	20.43 ± 0.23^b^	19.08 ± 0.28^a^	19.92 ± 0.21^b^	19.98 ± 0.17^b^	19.79 ± 0.25^b^
Ash	5.23 ± 0.01^a^	5.43 ± 0.02^b^	5.60 ± 0.02^c^	5.37 ± 0.01^b^	5.42 ± 0.04^b^
Crude fat	2.29 ± 0.03^b^	2.61 ± 0.04^d^	2.25 ± 0.02^b^	2.39 ± 0.02^c^	2.01 ± 0.02^a^
Crude protein	89.47 ± 0.25^c^	90.38 ± 0.15^d^	89.73 ± 0.38^cd^	88.63 ± 0.18^b^	86.32 ± 0.16^a^

The different lowercase letters, e.g., a, b, c, and d, have the meaning of significant differences (*p* < 0.05) from each other. The analyzed data are presented as the “Means ± SE.”

#### 3.5.2 Antioxidant Capacity in *Cynoglossus semilaevis* Muscles

Data on the anti-oxidative responses in the muscles of *C. semilaevis* under distinct diets-feeding are shown in Table 5. Significant differences were detected in SOD, T-AOC, CAT, MDA, and LPO activities among the treatments (*p* < 0.05). Concretely, no significant difference was detected in SOD between H0 and H100 groups, their SOD activities were greatly lower than that in the H25 and H75 groups. Meanwhile, the lowest level of SOD was measured in the H50 group. For the T-AOC, the smallest activity in the H25 *C. semilaevis* muscles was detected, their higher levels were analyzed in the H75, H100, and H0 groups, and yet less than that in the H50 group. The CAT activity in *C. semilaevis* muscles prominently reduced along with the rise in the DBLM content in feeding diets (*p* < 0.05). The MDA levels showed a markedly downward trend to the minimal activity (in the H50 group) at first, and then significantly increased to the greatest activity in the H100 group (*p* < 0.05). No significant differences in the LPO were shown between the H0 and H25 groups or between the H75 and H100 groups, but their activities were all significantly greater than that in the H50 group (Table 5).

**TABLE 5:** Antioxidation of *C. semilaevis* fed with different diets formulated with levels of DBLM (*n* = 4).

	H0	H25	H50	H75	H100
SOD	51.60 ± 0.10^b^	55.93 ± 0.09^c^	45.72 ± 0.23^a^	56.84 ± 0.06^d^	51.53 ± 0.11^b^
T-AOC	0.42 ± 0.00^c^	0.40 ± 0.00^a^	0.44 ± 0.00^d^	0.41 ± 0.00^b^	0.41 ± 0.00 ^ab^
CAT	6.64 ± 0.07^d^	3.77 ± 0.01^c^	3.62 ± 0.02^c^	3.19 ± 0.00^b^	2.83 ± 0.09^a^
MDA	2.78 ± 0.02^d^	2.67 ± 0.02^c^	2.47 ± 0.00^a^	2.61 ± 0.01^b^	5.64 ± 0.01^e^
LPO	0.23 ± 0.01^b^	0.22 ± 0.00^b^	0.08 ± 0.00^a^	0.28 ± 0.00^c^	0.28 ± 0.00^c^

Superoxide dismutase (SOD, U/mgprot), total antioxidant capacity (T-AOC, U/mL), malondialdehyde (MDA, mmol/L), catalase activity (CAT, U/mgprot), and lipid peroxide (LPO, mmol/L). The data were expressed by “mean ± SE.” Different lowercase letters mean the significant differences among the five experimental groups (*p* < 0.05).

#### 3.5.3 Histological Observation of *Cynoglossus semilaevis* Muscles

**FIGURE 4:**
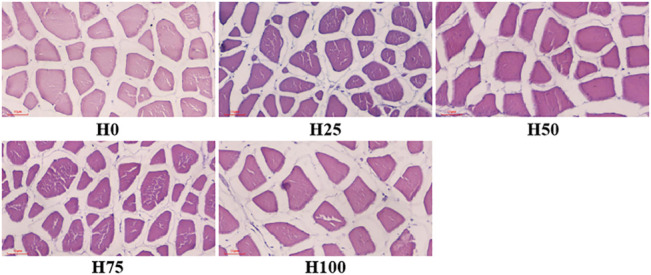
Cross sections of *C. semilaevis* muscles from the five experimental groups. Cross sections of *C. semilaevis* muscle tissue (×200 times) with structural organization. The red scale bar in the lower-left represents 50 μm and applies to all panels. The muscle fibers were stained with H&E.

**FIGURE 5:**
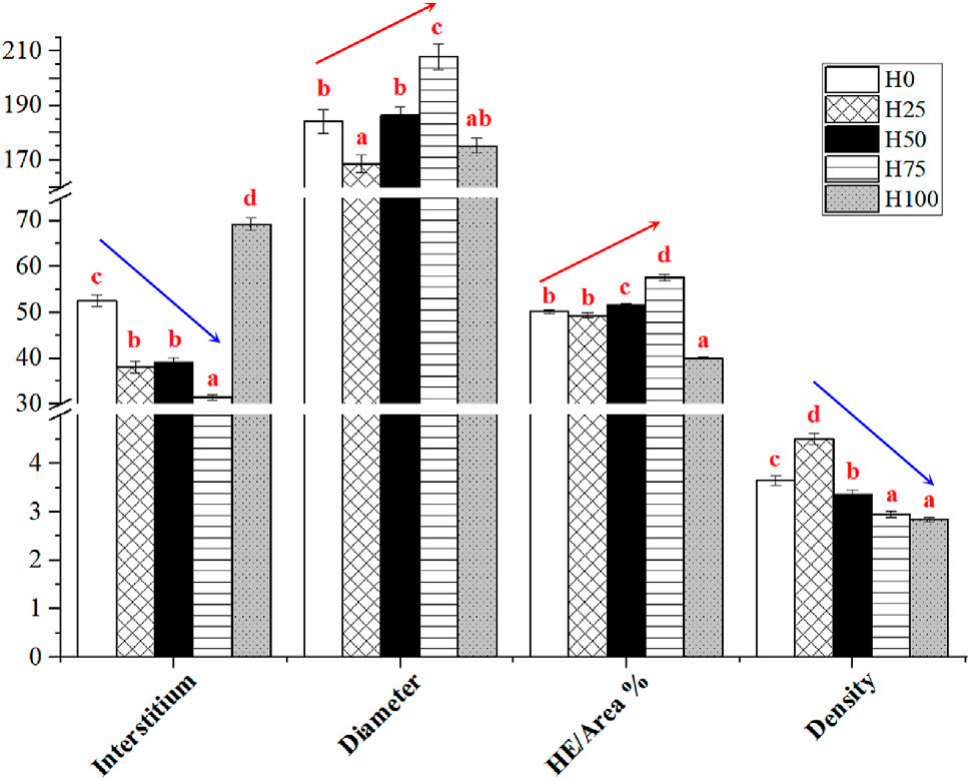
Quantitative results of the effect of DBLM diets on muscle fiber characteristics of *C. semilaevis.* Interstitium (Unit: μm), the distance between muscle fibers; diameter (Unit: μm), myofiber diameter; HE/area (%), the area of muscle fibers stained with H&E as the percentages of the area of the selected views; density, the numbers of myofibers within the area (*r* = 250 μm). The different lowercase letters indicate the significant differences among the five experimental groups (*p* < 0.05). The arrows represent the trends of indicators among the treatments. Data are presented as the “Mean ± SE.”

The histological changes were recorded using diameter and density of muscle fibers, interstitium between myofibers through the observation of cross sections of *C. semilaevis* muscles (Figures 4, 5). First, the distances between myofibers significantly reduced in the H25, H50, and H75 groups compared to the H0, but the maximum gap was detected in the H100 group (*p* < 0.05). There was an obvious downward trend in the density of muscle fibers as the increasing levels of DBLM in feeding diets. There were about 4, 5, 3, 2, and 2 fibers per unit area (*r* = 250 μm). It was also found that the percentage of muscle fiber area in the H75 group was significantly larger than that in other groups, whilst lowest in the H100 group (*p* < 0.05). Furthermore, no significant difference was found in the muscle fiber diameter among the H0, H50, and H100 groups, whose values were significantly larger than the H25 group’s, and smaller than that in the H75 *C. semilaevis* muscles.

#### 3.5.4 Water Holding Capacity of *Cynoglossus semilaevis* Muscles From Different Groups

**FIGURE 6:**
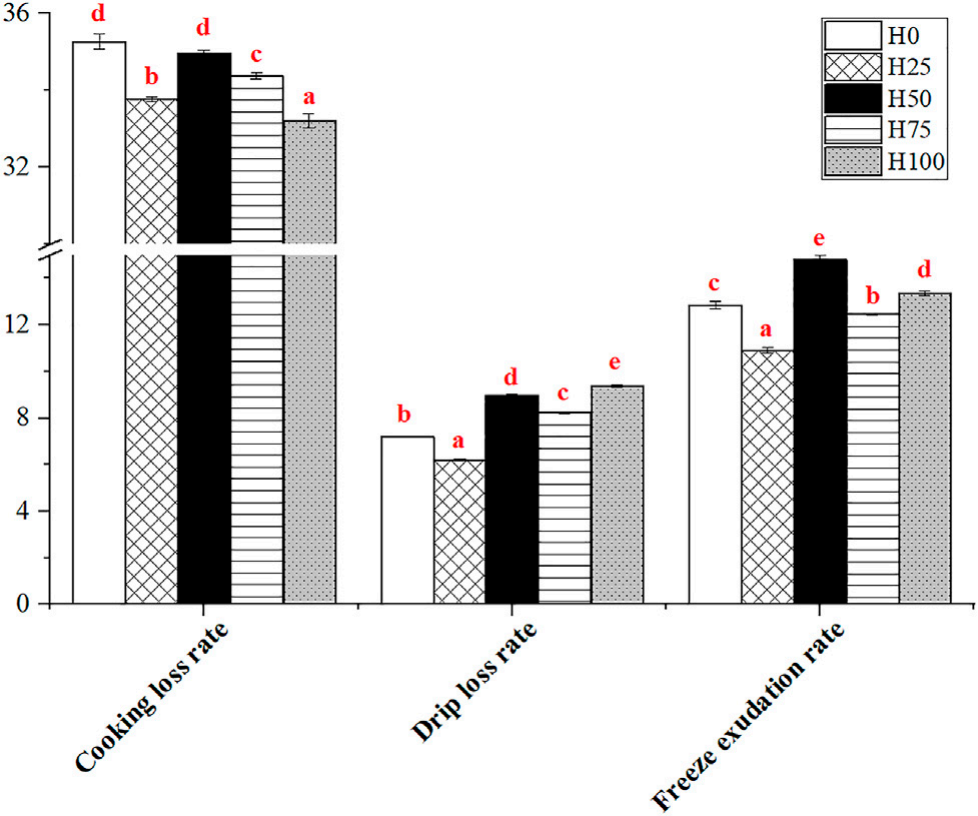
Effects of levels of DBLM diets on water holding capacity of *C. semilaevis* muscles (*n* = 12). The different lowercase letters mean the significant differences among groups (*p* < 0.05). Data are presented as the “Mean ± SE.”

The effect of levels of dietary DBLM on muscular water holding capacity was explained by the indexes, including the cooking loss rate (CLR), drip loss rate (DLR), and freeze exudation rate (FER). The CLR, DLR, and FER of *C. semilaevis* muscles were significantly influenced by the 5 dietary treatments (Figure 6). Meanwhile, these three indicators showed a similar change rule among the groups. The CLR, DLR, and FER of H0 *C. semilaevis* muscles were all significantly stronger than those of the H25 fish samples (*p* < 0.05), and almost the same or less than those in the H50 group. The three indexes were markedly reduced in the H75 group, but their levels were still higher than those in the H25 group (*p* < 0.05). The highest and lowest levels of CLR, DLR, and FER were detected in H0 and H100 groups, H100 and H25 groups, H25 and H50 groups, respectively.

## 4 Discussion

### 4.1 Differences in the Growth of *Cynoglossus semilaevis* Induced by DBLM Diet Feeding

In the present study, *C. semilaevis* fed with diets replaced 25% FM with DBLM showed significant improvements in weight gain rate (WGR), specific growth rate (SGR), condition factor (ConF), and survival rate (SR) compared to the control group, demonstrating the positive influence of supplemented lower level of DBLM on the growth of *C. semilaevis*. This conclusion has been confirmed in the study of the other species. For example, dietary replacement of FM by 25% insect powder, such as black soldier fly larvae meal (BLM), defatted BLM (DBLM), or *Tenebrio molitor* larvae meal, had no side effect on the growth performances of *O. mykiss* (Renna et al., 2017), *Litopenaeus vannamei* (Wang et al., 2021), *Clarias gariepinus* (Adeoye et al., 2020), and *Sparus aurata* (Piccolo et al., 2017). However, the level of insect meal replacing FM exceeded 50% and commonly resulted in a significant decline in the growth of fed fish. The dietary replacement of FM by 80% DBLM reduced the growth of *L. vannamei* (Wang et al., 2021), 100% BLM significantly restrained the SGR of *C. gariepinus* (Adeoye et al., 2020). In current work, the significantly reduced WGR, SGR, and ConF were found in the H50 group, and their lowest results were all detected in the H75 group, accompanied by the increases in feed conversion ratio (FCR) and feed intake (FI). It is the same as the results in *Ictalurus punctatus* (Yildirim-Aksoy et al., 2020). More specifically, the final weight gain of *I. punctatus* significantly increased when the feeding diets contained BLM at levels from 100 to 300 g/kg. *I. punctatus* fed diets without BLM, and with 300 g/kg BLM, showed the lowest and highest FI, respectively. The protein efficiency ratio (PER), however, was significantly lower in *I. punctatus* fed BLM at levels of 200 g/kg and higher compared to the FM diet (Yildirim-Aksoy et al., 2020). However, the research on *Dicentrarchus labrax* found that the dietary FM partially replaced by BLM showed no influences not only in growth performances of *D. labrax* but also in the feed utilization (Magalhães et al., 2017). Basto’s study found that it is feasible to substitute FM by defatted *T. molitor* larvae meal up to 80% without any significant effect on the growth of *D. labrax* (Basto et al., 2021). In *S. aurata* study testified that the more the replacement of FM by *T. molitor* larvae meal in diets, the less the FCR (Piccolo et al., 2017). The above diverse results could be due to the differences in fish or insect species, the different developmental stages of insects, as well as the varied processing methods for insects (Tschirner and Simon, 2015; Renna et al., 2017; English et al., 2021). In this study, the growth performances and feed utilization indices were observed as the largest or optimal values in the H25 group. Hence, the DBLM could replace 25% FM without any negative effects on the growth and feed utilization of *C. semilaevis*.

### 4.2 Effects of the Levels of Dietary DBLM on the Serum Biochemical Parameters of *Cynoglossus semilaevis*

Serum biochemical parameters are often used as a sensitive biomarker for confirming the aquatic product quality and for monitoring any changes in aquaculture in many fish species (Polakof et al., 2012). Currently, feeding *C. semilaevis* with DBLM diets brought the concentrations of serum T-CHO and TG down relative to the H0 group, which indicate that the more dietary DBLM supplied in diets could cut down the lipid level and fat accumulation in farmed fish. It is similar to the results in *D. labrax* (Magalhães et al., 2017), *C. carpio* (Li et al., 2017), *Lateolabrax japonicus* (Wang et al., 2019). These studies tend to claim that using DBLM instead of FM in feeding diets could effectively decrease the T-CHO and TG concentrations, meanwhile, relieve the fat deposition in the fish body that is exposed in the process of long-term feeding with FM feeds (Zhao et al., 2018). The higher content of chitosan in insects might be one of the important reasons which lead to the benefits abovementioned (Xia et al., 2011; Mohan et al., 2020). Another study demonstrates that the dietary inclusion of medium-chain fatty acid (MCFA) also resulted in decreased fat deposition in the tissues with improved growth of fish, and the MCFA is particularly high in black soldier fly larvae (Li S. et al., 2016).

The ALB, GLO, ALT, and AST are the available and sensitive indicators for the identification of hepatocyte injury and liver necrosis (Sheikhzadeh et al., 2012). Accordingly, the activities of serum GLO, AST, and ALT in DBLM diets-feeding groups (including H25, H50, H75, and H100 groups) were significantly higher than those in the H0 group, as well as the lower levels of ALB in DBLM diets-feeding groups. They all mean that abnormal liver function is likely to occur when *C. semilaevis* fed with DBLM diets (Lu et al., 2020). Whereas, no inflammation was found in the *D. rerio* liver fed with a diet supplemented with 50% BLM (Zarantoniello et al., 2018). Therefore, we boldly assume that the varied processing methods for insects also have quite different effects on the physiological and biochemical parameters of fed fish.

### 4.3 Differences in Digestive Enzyme Activity and Intestinal Microstructure Among Groups

The activity of intestinal digestive enzymes adapts to the changes in diets, also reflects the dietary influence on the feed utilization and growth performance of fish (Li et al., 2017; Zhang H. et al., 2020). The FCR and FI, as well as the activities of amylase, lipase, and trypsin were, examined higher even highest levels in the H75 *C. semilaevis*, with the smallest PER, WGR, SGR, and SR. Correspondingly, the H25 *C. semilaevis* exhibited the greatest PER, WGR, SGR, ConF, as well as the lowest FCR, FI and digestive enzyme activities. However, the negative correlation between growth performances and digestive enzyme activity is rarely reported in the investigation on the applications of BLM or DBLM in other species. Most studies suggested that the DBLM or BLM as an alternative protein ingredient had no significant effects on the activity of digestive enzymes, e.g., in *C. carpio* (Li et al., 2017), *L. vannamei* (Wang et al., 2021), *L. japonicus* (Wang et al., 2019) and *Micropterus salmoides* (Xu et al., 2021). The decreased activities of intestinal trypsin and lipase in *C. semilaevis* firstl, then enhanced with the increase in the addition level of DBLM. A similar result has been confirmed in *D. labrax* that increasing levels of DBLM in diets lowered the activities of intestinal trypsin and lipase (Lu et al., 2020). Because the different experimental diets in our study were isonitrogenous and isolipid, the alterations in digestive enzyme activities of *C. semilaevis* were not caused by the differences in the nutritional composition of feeding diets. The specific reason needs to be further verified.

The intestinal morphology (such as LM, CM, CE, ML, and LP) is valuable for the assessment of the gut health of fish (Yu et al., 2020). Accordingly, the significant improvements in LM, CM, CE, ML, and LP were found in the H25 group, whereas they were evidently reduced in the H50, H75, and H100 groups by a DBLM level-dependent manner. Ordinarily, the thinning of the intestinal lining is beneficial in increasing the absorption of nutrients which leads to optimize utilization and resulting in better growth (Cetingul et al., 2015; Wang et al., 2019). Therefore, the decreased thickness of CE, LP, CM, LM, and the length of microvilli, indicated that replacing FM with DBLM is helpful in improving the digestion and absorption of nutrients by *C. semilaevis*. However, it is worth noting that the substitution of FM increased up to 50%, accompanied by intestinal structural damage, such as splits of serosa and intestinal villus. In addition, the observations in intestinal morphology attributed to the varied contents of chitin in our five different feeding diets (Li Q. P. et al., 2016). Chitin was reported to decrease the thickness of intestinal wall and the length of microvilli (Li Q. P. et al., 2016).

### 4.4 Effects of Different Levels of DBLM on Meat Quality Characteristics of *Cynoglossus semilaevis*

The antioxidant system is the most important defense line of the body against oxidative damage (Xu et al., 2022). We found that superoxide dismutase (SOD) and catalase (CAT) declined significantly in the muscles of *C. semilaevis*, as the proportion of DBLM in the diets increased up to 75%. The data means that replaced level of less than 75% could boost the antioxidant capacity of *C. semilaevis* muscles, while the capacity would be inhibited if the level of replacing FM with DBLM was further increased. This situation was in accordance with Wang et al. (2019) who found that SOD activities in *L. japonicus* serum produced a similar trend when using DBLM as an alternative protein ingredient in diets. As mentioned previously, the more insect powder used, the more chitin contained, which is reported to have antioxidants, immune enhancement and other biological functions (Benhabiles et al., 2012; Mengíbar et al., 2013; Zou et al., 2016; Naveed et al., 2019). However, we need to pay attention to the negative effect of the excessive use of DBLM. For instance, substituting the FM with DBLM over 75% resulted in an oxidative damage in *C. semilaevis* muscles, characterized by increasing the products of lipid peroxidation, such as MDA and LPO. This conclusion was supported by the previous findings in *D. rerio* (Zarantoniello et al., 2018) and *L. japonicus* (Wang et al., 2019).

In addition, the contents of crude fat (CF), crude protein (CP), and ash in *C. semilaevis* fillets were not affected by dietary DBLM levels, without changes in moisture and dry matter (DM). It is similar to the results reported in *Paralichthys olivaceus*, which revealed that no changes were evident in moisture and ash, but the CF and CP contents showed a significant decreasing trend with the rise in dietary insect powder (Jeong et al., 2021b). Belforti et al. (2016) found that the CP in fillets rose significantly with the increased DBLM in diets. Water holding capacity, an important index to evaluate the meat quality, is reflected by DLR, CLR, and FER (Zhang L. et al., 2020). There was research illustrated that water holding capacity poses positive correlations with the density of myofibers and the CP in fillets (Periago et al., 2005; Cheng et al., 2014). Accordingly, the largest density of myofibers, most abundant CP, and the lowest DLR, CLR, FER were all detected in the H25 *C. semilaevis* fillets. Consequently, the water holding capacity of H25 *C. semilaevis* muscles was the best among the groups. The study on *O. mykiss* came to a same conclusion (Caimi et al., 2021).

## 5 Conclusion

We conducted a comprehensive study to investigate the effects of different levels of DBLM on the growth, digestive function, and flesh quality of *C. semilaevis*. The alterations in all testing indices are deeply intertwined and eventually give rise to the differences in the muscle growth, digestion, and muscle quality among groups. Concretely, *C. semilaevis* fed with diets replaced by 25% FM with DBLM showed significant improvements in WGR, SGR, ConF, and SR compared to the H0 group, demonstrating the positive influence of supplemented lower level of DBLM on the growth of *C. semilaevis*, accompanied with the decline in feed conversion ratio (FCR) and feed intake (FI). Feeding *C. semilaevis* with DBLM diets could cut down the lipid level and relieve the fat deposition in the fish body by downregulating the concentrations of serum T-CHO and TG. Furthermore, the thinning of the intestinal lining, found in the H50, H75, and H100 groups, is beneficial in enhancing the absorption of nutrients leading to optimize the utilization and resulting in better growth. However, the proportion of DBLM replacing FM exceeded 50% also caused intestinal histopathological damage. In addition, the negative effects (e.g., splits of serosa and intestinal villus, increasing the products of lipid peroxidation, and abnormal liver function) of excessive use of DBLM (>75%) were also proved in our study, though the significantly improved antioxidant capacity was detected in the H75 *C. semilaevis* muscles. In addition, the largest density of myofibers, most abundant of CP, and lowest DLR, CLR, FER were all detected in the H25 *C. semilaevis* fillets. By inference, the water holding capacity was the best in the H25 group.

In conclusion, the application of *H. illucens* in aquatic feeds to replace a fish meal with a lower level for culturing *C. semilaevis* could improve feed utilization, promote growth performances, enhance the digestive ability, and obtain better quality of farmed fish. This research consists of an important step toward looking for an efficiently alternative protein source of fish meal, meeting consumers’ demand for high-quality, safe, and healthy aquatic products.

## Data Availability

The raw data supporting the conclusion of this article will be made available by the authors, without undue reservation.

## References

[B1] Adeoye A. A. Akegbejo‐Samsons Y. Fawole F. J. Davies S. J. (2020). Preliminary Assessment of Black oldier Fly ( Hermetia Illucens ) Larval Meal in the Diet of African Catfish ( *Clarias gariepinus* ): Impact on Growth, Body index, and Hematological Parameters. J. World Aquacult Soc. 51, 1024–1033. 10.1111/jwas.12691

[B2] AOAC (2012). Official Methods of Analysis (19^th^ Edition), Chapter 4, Association of Official Analytical Chemists . Gaithersburgh, USA: AOAC International, 9–13.

[B3] Basto A. Calduch-Giner J. Oliveira B. Petit L. Sá T. Maia M. R. G. (2021). The Use of Defatted *Tenebrio molitor* Larvae Meal as a Main Protein Source Is Supported in European Sea Bass (*Dicentrarchus labrax*) by Data on Growth Performance, Lipid Metabolism, and Flesh Quality. Front. Physiol. 12 (12), 659567. 10.3389/fphys.2021.659567 33967831 PMC8104126

[B4] Belforti M. Gai F. Lussiana C. Renna M. Malfatto V. Rotolo L. (2016). Tenebrio MolitorMeal in Rainbow Trout (Oncorhynchus Mykiss) Diets: Effects on Animal Performance, Nutrient Digestibility and Chemical Composition of Fillets. Ital. J. Anim. Sci. 14 (4), 4170. 10.4081/ijas.2015.4170

[B5] Benhabiles M. S. Salah R. Lounici H. Drouiche N. Goosen M. F. A. Mameri N. (2012). Antibacterial Activity of Chitin, Chitosan and its Oligomers Prepared from Shrimp Shell Waste. Food Hydrocolloids 29 (1), 48–56. 10.1016/j.foodhyd.2012.02.013

[B6] Caimi C. Biasato I. Chemello G. Oddon S. B. Lussiana C. Malfatto V. M. (2021). Dietary Inclusion of a Partially Defatted Black Soldier Fly (*Hermetia Illucens*) Larva Meal in Low Fishmeal-Based Diets for Rainbow trout (*Oncorhynchus mykiss*). J. Anim. Sci Biotechnol 12 (1), 50. 10.1186/s40104-021-00575-1 33858519 PMC8050899

[B7] Cavrois-Rogacki T. Leeming D. Lopez P. M. Davie A. Migaud H. (2022). Plant-based Protein Ingredients Can Successfully Replace Fish Meal in the Diet of Ballan Wrasse (*Labrus Bergylta*) Juveniles. Aquaculture 546, 737419. 10.1016/j.aquaculture.2021.737419

[B8] Cetingul I. S. Rahman A. Ulucan A. Keles H. Bayram I. Uyarlar C. (2015). Effect of Mentha Piperita on Some Morphological Characteristics of Intestine in Japanese Quails (*Coturnix coturnix Japonica*). Archiva Zootechnica 18 (2), 53–60.

[B9] Cheng J.-H. Sun D.-W. Han Z. Zeng X.-A. (2014). Texture and Structure Measurements and Analyses for Evaluation of Fish and Fillet Freshness Quality: A Review. Compr. Rev. Food Sci. Food Saf. 13 (1), 52–61. 10.1111/1541-4337.12043 33412693

[B10] Crane D. P. Ogle D. H. Shoup D. E. (2020). Use and Misuse of a Common Growth Metric: Guidance for Appropriately Calculating and Reporting Specific Growth Rate. Rev. Aquac. 12 (3), 1542–1547. 10.1111/raq.12396

[B11] English G. Wanger G. Colombo S. M. (2021). A Review of Advancements in Black Soldier Fly (*Hermetia Illucens*) Production for Dietary Inclusion in Salmonid Feeds. J. Agric. Food Res. 5, 100164. 10.1016/j.jafr.2021.100164

[B12] Folch J. Lees M. Stanley G. H. S. (1957). A Simple Method for the Isolation and Purification of Total Lipides from Animal Tissues. J. Biol. Chem. 226 (1), 497–509. 10.1016/S0021-9258(18)64849-5 13428781

[B13] Hardy R. W. (2010). Utilization of Plant Proteins in Fish Diets: Effects of Global Demand and Supplies of Fishmeal. Aquac. Res. 41 (5), 770–776. 10.1111/j.1365-2109.2009.02349.x

[B14] Jeong S.-M. Khosravi S. Yoon K.-Y. Kim K.-W. Lee B.-J. Hur S.-W. (2021b). Mealworm, *Tenebrio molitor*, as a Feed Ingredient for Juvenile Olive Flounder, *Paralichthys olivaceus* . Aquac. Rep. 20, 100747. 10.1016/j.aqrep.2021.100747

[B15] Jeong S. M. Khosravi S. Mauliasari I. R. Lee B. J. You S. G. Lee S. M. (2021a). Nutritional Evaluation of Cricket, Gryllus Bimaculatus , Meal as Fish Meal Substitute for Olive Flounder, *Paralichthys olivaceus* , Juveniles. J. World Aquac. Soc. 52, 859–880. 10.1111/jwas.12790

[B16] Lanes C. F. C. Pedron F. A. Bergamin G. T. Bitencourt A. L. Dorneles B. E. R. Villanova J. C. V. (2021). Black Soldier Fly (*Hermetia Illucens*) Larvae and Prepupae Defatted Meals in Diets for Zebrafish (*Danio rerio*). Animals 11 (3), 720. 10.3390/ani11030720 33800826 PMC7999764

[B17] Lee D.-H. Chu K.-B. Kang H.-J. Lee S.-H. Quan F.-S. (2020). Peptides in the Hemolymph of *Hermetia Illucens* Larvae Completely Inhibit the Growth of *Klebsiella Pneumonia In Vitro* and *In Vivo* . J. Asia-Pacific Entomol. 23, 36–43. 10.1016/j.aspen.2019.10.004

[B18] Li Q. P. Gooneratne S. R. Wang R. L. Zhang R. An L. L. Chen J. J. (2016a). Effect of Different Molecular Weight of Chitosans on Performance and Lipid Metabolism in Chicken. Anim. Feed Sci. Tech. 211, 174–180. 10.1016/j.anifeedsci.2015.11.013

[B19] Li S. Ji H. Zhang B. Tian J. Zhou J. Yu H. (2016b). Influence of Black Soldier Fly ( Hermetia Illucens ) Larvae Oil on Growth Performance, Body Composition, Tissue Fatty Acid Composition and Lipid Deposition in Juvenile Jian Carp ( *Cyprinus carpio* Var. Jian). Aquaculture 465, 43–52. 10.1016/j.aquaculture.2016.08.020

[B20] Li S. Ji H. Zhang B. Zhou J. Yu H. (2017). Defatted Black Soldier Fly ( Hermetia Illucens ) Larvae Meal in Diets for Juvenile Jian Carp ( *Cyprinus carpio* Var. Jian): Growth Performance, Antioxidant Enzyme Activities, Digestive Enzyme Activities, Intestine and Hepatopancreas Histological Structure. Aquaculture 477, 62–70. 10.1016/j.aquaculture.2017.04.015

[B21] Lin G. Gao D. Lu J. Sun X. (2021). Transcriptome Profiling Reveals the Sexual Dimorphism of Gene Expression Patterns during Gonad Differentiation in the Half-Smooth Tongue Sole (*Cynoglossus Semilaevis*). Mar. Biotechnol. 23 (1), 18–30. 10.1007/s10126-020-09996-x 32996005

[B22] Lu R. Chen Y. Yu W. Lin M. Yang G. Qin C. (2020). Defatted Black Soldier Fly (*Hermetia Illucens*) Larvae Meal Can Replace Soybean Meal in Juvenile Grass Carp (*Ctenopharyngodon idellus*) Diets. Aquac. Rep. 18, 100520. 10.1016/j.aqrep.2020.100520

[B23] Luther P. K. Munro P. M. G. Squire J. M. (1995). Muscle Ultrastructure in the Teleost Fish. Micron 26, 431–459. 10.1016/0968-4328(95)00015-1

[B24] Magalhães R. Sánchez-López A. Leal R. S. Martínez-Llorens S. Oliva-Teles A. Peres H. (2017). Black Soldier Fly (*Hermetia Illucens*) Pre-pupae Meal as a Fish Meal Replacement in Diets for European Seabass (*Dicentrarchus labrax*). Aquaculture 476, 79–85. 10.1016/j.aquaculture.2017.04.021

[B25] Mengíbar M. Mateos-Aparicio I. Miralles B. Heras A. (2013). Influence of the Physico-Chemical Characteristics of Chito-Oligosaccharides (COS) on Antioxidant Activity. Carbohydr. Polym. 97 (2), 776–782. 10.1016/j.carbpol.2013.05.035 23911515

[B26] Mohan K. Ganesan A. R. Muralisankar T. Jayakumar R. Sathishkumar P. Uthayakumar V. (2020). Recent Insights into the Extraction, Characterization, and Bioactivities of Chitin and Chitosan from Insects. Trends Food Sci. Tech. 105, 17–42. 10.1016/j.tifs.2020.08.016 PMC7471941 32901176

[B27] Naveed M. Phil L. Sohail M. Hasnat M. Baig M. M. F. A. Ihsan A. U. (2019). Chitosan Oligosaccharide (COS): An Overview. Int. J. Biol. Macromolecules 129, 827–843. 10.1016/j.ijbiomac.2019.01.192 30708011

[B28] Nogales-Mérida S. Gobbi P. Jozefiak D. Mazurkiewicz J. Dudek K. Rawski M. (2019). Insect Meals in Fish Nutrition. Rev. Aquac. 11, 1080–1103.

[B29] Periago M. J. Ayala M. D. López-Albors O. Abdel I. Martínez C. García-Alcázar A. (2005). Muscle Cellularity and Flesh Quality of Wild and Farmed Sea Bass. Dicentrarchus labrax L. Aquac. 249 (1-4), 175–188. 10.1016/j.aquaculture.2005.02.047

[B30] Piccolo G. Iaconisi V. Marono S. Gasco L. Loponte R. Nizza S. (2017). Effect of *Tenebrio molitor* Larvae Meal on Growth Performance, *In Vivo* Nutrients Digestibility, Somatic and Marketable Indexes of Gilthead Sea Bream (*Sparus Aurata*). Anim. Feed Sci. Tech. 226, 12–20. 10.1016/j.anifeedsci.2017.02.007

[B31] Polakof S. Panserat S. Soengas J. L. Moon T. W. (2012). Glucose Metabolism in Fish: a Review. J. Comp. Physiol. B 182, 1015–1045. 10.1007/s00360-012-0658-7 22476584

[B32] Raksasat R. Lim J. W. Kiatkittipong W. Kiatkittipong K. Ho Y. C. Lam M. K. (2020). A Review of Organic Waste Enrichment for Inducing Palatability of Black Soldier Fly Larvae: Wastes to Valuable Resources. Environ. Pollut. 267, 115488. 10.1016/j.envpol.2020.115488 32891050

[B33] Rasmussen R. S. Ostenfeld T. H. (2000). Influence of Growth Rate on white Muscle Dynamics in Rainbow trout and brook trout. J. Fish Biol. 56, 1548–1552. 10.1111/j.1095-8649.2000.tb02164.x

[B34] Rawski M. Mazurkiewicz J. Kierończyk B. Józefiak D. (2020). Black Soldier Fly Full-Fat Larvae Meal as an Alternative to Fish Meal and Fish Oil in Siberian sturgeon Nutrition: The Effects on Physical Properties of the Feed, Animal Growth Performance, and Feed Acceptance and Utilization. Animals 10, 2119. 10.3390/ani10112119 PMC7697048 33203187

[B35] Renna M. Schiavone A. Gai F. Dabbou S. Lussiana C. Malfatto V. (2017). Evaluation of the Suitability of a Partially Defatted Black Soldier Fly (*Hermetia Illucens* L.) Larvae Meal as Ingredient for Rainbow trout (*Oncorhynchus mykiss* Walbaum) Diets. J. Anim. Sci Biotechnol 8, 57. 10.1186/s40104-017-0191-3 28680591 PMC5494141

[B36] Sheikhzadeh N. Tayefi-Nasrabadi H. Khani Oushani A. Najafi Enferadi M. H. (2012). Effects of *Haematococcus pluvialis* Supplementation on Antioxidant System and Metabolism in Rainbow trout (*Oncorhynchus mykiss*). Fish. Physiol. Biochem. 38 (2), 413–419. 10.1007/s10695-011-9519-7 21695482

[B37] Shi X. Li D. Zhuang P. Nie F. Long L. (2006). Comparative Blood Biochemistry of Amur sturgeon, Acipenser Schrenckii, and Chinese Surgeon, Acipenser Sinensis. Fish. Physiol. Biochem. 32, 63–66. 10.1007/s10695-006-7134-9 20035480

[B38] Smets R. Verbinnen B. Van De Voorde I. Aerts G. Claes J. Van Der Borght M. (2020). Sequential Extraction and Characterisation of Lipids, Proteins, and Chitin from Black Soldier Fly (*Hermetia Illucens*) Larvae, Prepupae, and Pupae. Waste Biomass Valor. 11, 6455–6466. 10.1007/s12649-019-00924-2

[B39] Spranghers T. Ottoboni M. Klootwijk C. Ovyn A. Deboosere S. De Meulenaer B. (2017). Nutritional Composition of Black Soldier Fly (*Hermetia Illucens*) Prepupae Reared on Different Organic Waste Substrates. J. Sci. Food Agric. 97, 2594–2600. 10.1002/jsfa.8081 27734508

[B40] Stejskal V. Tran H. Q. Prokesova M. Gebauer T. Giang P. T. Gai F. (2020). Partially Defatted *Hermetia Illucens* Larva Meal in Diet of Eurasian Perch (*Perca fluviatilis*) Juveniles. Animals 10 (10), 1876. 10.3390/ani10101876 PMC7602402 33066664

[B41] Tacon A. G. J. Metian M. (2008). Global Overview on the Use of Fish Meal and Fish Oil in Industrially Compounded Aquafeeds: Trends and Future Prospects. Aquaculture 285 (1-4), 146–158. 10.1016/j.aquaculture.2008.08.015

[B42] Takakuwa F. Tanabe R. Nomura S. Inui T. Yamada S. Biswas A. (2021). Availability of Black Soldier Fly Meal as an Alternative Protein Source to Fish Meal in Red Sea Bream (*Pagrus Major*, Temminck & Schlegel) Fingerling Diets. Aquac. Res. 00, 1–14. 10.1111/are.15550

[B43] Tippayadara N. Dawood M. A. O. Krutmuang P. Hoseinifar S. H. Doan H. V. Paolucci M. (2021). Replacement of Fish Meal by Black Soldier Fly (*Hermetia Illucens*) Larvae Meal: Effects on Growth, Haematology, and Skin Mucus Immunity of Nile tilapia, *Oreochromis niloticus* . Animals 11, 193. 10.3390/ani11010193 33467482 PMC7830215

[B44] Tschirner M. Simon A. (2015). Influence of Different Growing Substrates and Processing on the Nutrient Composition of Black Soldier Fly Larvae Destined for Animal Feed. J. Insects as Food Feed 1 (4), 249–259. 10.3920/jiff2014.0008

[B45] Urán P. A. Schrama J. W. Rombout J. H. W. M. Obach A. Jensen L. Koppe W. (2008). Soybean Meal-Induced Enteritis in Atlantic salmon (*Salmo salar* L.) at Different Temperatures. Aquac. Nutr. 14, 324–330. 10.1111/j.1365-2095.2007.00534.x

[B46] Valente L. M. P. Moutou K. A. Conceição L. E. C. Engrola S. Fernandes J. M. O. Johnston I. A. (2013). What Determines Growth Potential and Juvenile Quality of Farmed Fish Species? Rev. Aquacult. 5, 168–193. 10.1111/raq.12020

[B47] Wang G. Peng K. Hu J. Mo W. Wei Z. Huang Y. (2021). Evaluation of Defatted Hermetia Illucens Larvae Meal for Litopenaeus Vannamei : Effects on Growth Performance, Nutrition Retention, Antioxidant and Immune Response, Digestive Enzyme Activity and Hepatic Morphology. Aquacult Nutr. 27 (4), 986–997. 10.1111/anu.13240

[B48] Wang G. Peng K. Hu J. Yi C. Chen X. Wu H. (2019). Evaluation of Defatted Black Soldier Fly (*Hermetia Illucens* L.) Larvae Meal as an Alternative Protein Ingredient for Juvenile Japanese Seabass (*Lateolabrax Japonicus*) Diets. Aquaculture 507, 144–154. 10.1016/j.aquaculture.2019.04.023

[B49] Wang Y.-S. Shelomi M. (2017). Review of Black Soldier Fly (*Hermetia Illucens*) as Animal Feed and Human Food. Foods 6, 91. 10.3390/foods6100091 PMC5664030 29057841

[B50] Xia W. Liu P. Zhang J. Chen J. (2011). Biological Activities of Chitosan and Chitooligosaccharides. Food Hydrocolloids 25 (2), 170–179. 10.1016/j.foodhyd.2010.03.003

[B51] Xu F.-M. Hou S.-W. Wang G.-X. Gong J.-Y. Zhou L. Huang Y.-H. (2021). Effects of Zymolytic Black Soldier Fly (*Hermetia Illucens*) Pulp as Dietary Supplementation in Largemouth Bass (*Micropterus salmoides*). Aquac. Rep. 21, 100823. 10.1016/j.aqrep.2021.100823

[B52] Xu M. Chen X. Huang Z. Chen D. Li M. He J. (2022). Effects of Dietary Grape Seed Proanthocyanidin Extract Supplementation on Meat Quality, Muscle Fiber Characteristics and Antioxidant Capacity of Finishing Pigs. Food Chem. 367, 130781. 10.1016/j.foodchem.2021.130781 34391997

[B53] Xu X. X. Ji H. Yu H. B. Zhou J. S. (2020). Influence of Dietary Black Soldier Fly (Hermetia Illucens Linnaeus) Pulp on Growth Performance, Antioxidant Capacity and Intestinal Health of Juvenile Mirror Carp (*Cyprinus carpio* Var. Specularis). Aquac. Nutr. 00, 1–12. 10.1111/anu.13005

[B54] Yildirim-Aksoy M. Eljack R. Beck B. H. (2020). Nutritional Value of Frass from Black Soldier Fly Larvae, *Hermetia Illucens*, in a Channel Catfish, *Ictalurus punctatus*, Diet. Aquac. Nutr. 26 (3), 812–819. 10.1111/anu.13040

[B55] Yu L. Wen H. Jiang M. Wu F. Tian J. Lu X. (2020). Effects of Ferulic Acid on Intestinal Enzyme Activities, Morphology, Microbiome Composition of Genetically Improved Farmed tilapia (*Oreochromis niloticus*) Fed Oxidized Fish Oil. Aquaculture 528, 735543. 10.1016/j.aquaculture.2020.735543

[B56] Zar J. H. (1996). Biostatistical Analysis. 3 ^rd^ Edn. Prentice-Hall International. Hoboken, New Jersey.

[B57] Zarantoniello M. Bruni L. Randazzo B. Vargas A. Gioacchini G. Truzzi C. (2018). Partial Dietary Inclusion of *Hermetia Illucens* (Black Soldier Fly) Full-Fat Prepupae in Zebrafish Feed: Biometric, Histological, Biochemical, and Molecular Implications. Zebrafish 15 (5), 519–532. 10.1089/zeb.2018.1596 29912648

[B58] Zhang H. Ding Q. Wang A. Liu Y. Teame T. Ran C. (2020a). Effects of Dietary Sodium Acetate on Food Intake, Weight Gain, Intestinal Digestive Enzyme Activities, Energy Metabolism and Gut Microbiota in Cultured Fish: Zebrafish as a Model. Aquaculture 523, 735188. 10.1016/j.aquaculture.2020.735188

[B59] Zhang L. Huang H. Wang P. Xing T. Xu X. (2020b). Water-spraying Forced Ventilation during Holding Improves the Water Holding Capacity, Impedance, and Microstructure of Breast Meat from Summer-Transported Broiler Chickens. Poult. Sci. 99 (3), 1744–1749. 10.1016/j.psj.2019.10.077 32115041 PMC7587643

[B60] Zhao H. Xia J. Zhang X. He X. Li L. Tang R. (2018). Diet Affects Muscle Quality and Growth Traits of Grass Carp (*Ctenopharyngodon idellus*): A Comparison between Grass and Artificial Feed. Front. Physiol. 9, 283. 10.3389/fphys.2018.00283 29632496 PMC5879129

[B61] Zou P. Yang X. Wang J. Li Y. Yu H. Zhang Y. (2016). Advances in Characterisation and Biological Activities of Chitosan and Chitosan Oligosaccharides. Food Chem. 190, 1174–1181. 10.1016/j.foodchem.2015.06.076 26213092

